# A collaborative semantic-based provenance management platform for reproducibility

**DOI:** 10.7717/peerj-cs.921

**Published:** 2022-03-10

**Authors:** Sheeba Samuel, Birgitta König-Ries

**Affiliations:** 1 Michael Stifel Center Jena, Jena, Germany; 2 Heinz Nixdorf Chair for Distributed Information Systems, Friedrich-Schiller Universität Jena, Jena, Thuringia, Germany

**Keywords:** Provenance, Reproducibility, Research data management platform, Jupyter Notebooks, Scientific experiments, Ontology, Visualization, Semantic Web

## Abstract

Scientific data management plays a key role in the reproducibility of scientific results. To reproduce results, not only the results but also the data and steps of scientific experiments must be made findable, accessible, interoperable, and reusable. Tracking, managing, describing, and visualizing provenance helps in the understandability, reproducibility, and reuse of experiments for the scientific community. Current systems lack a link between the data, steps, and results from the computational and non-computational processes of an experiment. Such a link, however, is vital for the reproducibility of results. We present a novel solution for the end-to-end provenance management of scientific experiments. We provide a framework, CAESAR (CollAborative Environment for Scientific Analysis with Reproducibility), which allows scientists to capture, manage, query and visualize the complete path of a scientific experiment consisting of computational and non-computational data and steps in an interoperable way. CAESAR integrates the REPRODUCE-ME provenance model, extended from existing semantic web standards, to represent the whole picture of an experiment describing the path it took from its design to its result. ProvBook, an extension for Jupyter Notebooks, is developed and integrated into CAESAR to support computational reproducibility. We have applied and evaluated our contributions to a set of scientific experiments in microscopy research projects.

## Introduction

Reproducibility of results is vital in every field of science. The scientific community is interested in the results of experiments that are accessible, reproducible, and reusable. Recent surveys conducted among researchers show the existence of a problem in reproducing published results in different disciplines (Baker, 2016; Samuel & König-Ries, 2021). Recently, there is a rapidly growing awareness in scientific disciplines on the importance of reproducibility. As a consequence, measures are being taken to make the data used in the publications FAIR (**F**indable, **A**ccessible, **I**nteroperable, **R**eusable) (Wilkinson et al., 2016). However, this is too little too late: these measures are usually taken at the point in time when papers are being published. These measures do not include the management and description of several trials of an experiment, negative results from these trials, dependencies between the data and steps, *etc.* However, many challenges, particularly for data management, are faced by scientists much earlier in the scientific cycle. If they are not addressed properly and in a timely manner, they often make it impossible to provide truly FAIR data at the end. Therefore, we argue that scientists need support from the very beginning of an experiment in handling the potentially large amounts of heterogeneous research data and its derivation.

A key factor to support scientific reproducibility is the *provenance* information that tells about the origin or history of the data. Recording and analysis of provenance data of a scientific experiment play a vital role for scientists to know the methods and steps taken to generate the output, to reproduce own results or other scientist’s results (Taylor & Kuyatt, 1994). In addition to the preservation of data and results, the datasets and the metadata need to be collected and organized in a structured way from the beginning of the experiments. At the same time, information should be represented and expressed in an interoperable way so that scientists can understand the data and results. Therefore, we need to start addressing this issue at the stage when the data is created. Thus, scientific research data management needs to start at the earlier stage of the research lifecycle to play a vital role in this context.

In this paper, we aim to provide end-to-end provenance capture and management of scientific experiments to support reproducibility. To define our aim, we define the main research question, which structure the remainder of the article: How can we capture, represent, manage and visualize a complete path taken by a scientist in an experiment, including the computational and non-computational steps to derive a path towards experimental results? To address the research question, we create a conceptual model using semantic web technologies to describe a complete path of a scientific experiment. We design and develop a provenance-based semantic framework to populate this model, collect information about the experimental data and results along with the settings, runs, and execution environment and visualize them. The main contribution of this paper is the framework for the end-to-end provenance management of scientific experiments, called CAESAR (**C**oll**A**borative **E**nvironment for **S**cientific **A**nalysis with **R**eproducibility) integrated with ProvBook (Samuel & König-Ries, 2018b) and REPRODUCE-ME data model (Samuel, 2019a). CAESAR supports computational reproducibility using our tool ProvBook, which is designed and developed to capture, store, compare and track the provenance of results of different executions of Jupyter Notebooks. The complete path of a scientific experiment interlinking the computational and non-computational data and steps is semantically represented using the REPRODUCE-ME data model.

In the following sections, we provide a detailed description of our findings. We start with an overview of the current state-of-the-art (“Related Work”). We describe the experimental methodology used in the development of CAESAR (“Materials & Methods”). In the “Results”, we describe CAESAR and its main modules. We describe the evaluation strategies and results in the “Evaluation”. In the ‘Discussion”, we discuss the implications of our results and the limitations of our approach. We conclude the article by highlighting our major findings in the “Conclusion”.

## Related Work

Scientific data management plays a key role in knowledge discovery, data integration, and reuse. The preservation of digital objects has been studied for long in the digital preservation community. Some works give more importance to software and business process conservation (Mayer et al., 2012), while other works focus on scientific workflow preservation (Belhajjame et al., 2015). We focus our approach more on the data management solutions for scientific data, including images. Eliceiri et al. (2012) provide a list of biological imaging software tools. BisQue is an open-source, server-based software system that can store, display and analyze images (Kvilekval et al., 2010). OMERO, developed by the Open Microscopy Environment (OME), is another open-source data management platform for imaging metadata primarily for experimental biology (Allan et al., 2012). It has a plugin architecture with a rich set of features, including analyzing and modifying images. It supports over 140 image file formats using BIO-Formats (Linkert et al., 2010). OMERO and BisQue are the two closest solutions that meet our requirements in the context of scientific data management. A general approach to document experimental metadata is provided by the CEDAR workbench (Gonçalves et al., 2017). It is a metadata repository with a web-based tool that helps users to create metadata templates and fill in the metadata using those templates. However, these systems do not directly provide the features to fully capture, represent and visualize the complete path of a scientific experiment and support computational reproducibility and semantic integration.

Several tools have been developed to capture complete computational workflows to support reproducibility in the context of scientific workflows, scripts, and computational notebooks. Oliveira, Oliveira & Braganholo (2018) survey the current state of the art approaches and tools that support provenance data analysis for workflow-based computational experiments. Scientific Workflows, which are a complex set of data processes and computations, are constructed with the help of a Scientific Workflow Management System(SWfMS) (Deelman et al., 2005; Liu et al., 2015). Different SWfMSs have been developed for different uses cases and domains (Oinn et al., 2004; Altintas et al., 2004; Deelman et al., 2005; Scheidegger et al., 2008; Goecks, Nekrutenko & Taylor, 2010). Most SWfMSs provide provenance support by capturing the history of workflow executions. These systems focus on the computational steps of an experiment and do not link the results to the experimental metadata. Despite the provenance modules present in these systems, there are currently many challenges in the context of reproducibility of scientific workflows (Zhao et al., 2012; Cohen-Boulakia et al., 2017). Workflows created by different scientists are difficult for others to understand or re-run in a different environment, resulting in workflow decays (Zhao et al., 2012). The lack of interoperability between scientific workflows and the steep learning curve required by scientists are some of the limitations according to the study of different SWfMSs (Cohen-Boulakia et al., 2017). The Common Workflow Language (Amstutz et al., 2016) is an initiative to overcome the lack of interoperability of workflows. Though there is a learning curve associated with adopting workflow languages, this ongoing work aims to make computational methods reproducible, portable, maintainable, and shareable.

Many tools have been developed to capture the provenance of results from the scripts at different levels of granularity (Guo & Seltzer, 2012; Davison, 2012; Murta et al., 2014; McPhillips et al., 2015). Burrito (Guo & Seltzer, 2012) captures provenance at the operating system level and provides a user interface for documenting and annotating the provenance of non-computational processes. Carvalho, Belhajjame & Medeiros (2016) present an approach to convert scripts into reproducible Workflow Research Objects. However, it is a complex process that requires extensive involvement of scientists and curators with extensive knowledge of the workflow and script programming in every step of the conversion. The lack of documentation of computational experiments along with their results and the ability to reuse parts of code are some of the issues hindering reproducibility in script-based environments. In recent years, computational notebooks have gained widespread adoption because they enable computational reproducibility and allow users to share code along with documentation. Jupyter Notebook (Kluyver et al., 2016), which was formerly known as the IPython notebook, is a widely used computational notebook that provides an interactive environment supporting over 100 programming languages with millions of users around the world. Even though it supports reproducible research, recent studies by Rule, Tabard & Hollan (2018) and Pimentel et al. (2019) point out the need for provenance support in computational notebooks. Overwriting and re-execution of cells in any order can lead to the loss of results from previous trials. Some research works have attempted to track provenance from computational notebooks (Hoekstra, 2014; Pimentel et al., 2015; Carvalho et al., 2017; Koop & Patel, 2017; Kery & Myers, 2018; Petricek, Geddes & Sutton, 2018; Head et al., 2019; Wenskovitch et al., 2019; Wang et al., 2020; Macke et al., 2021). Pimentel et al. (2015) propose a mechanism to capture and analyze the provenance of python scripts inside IPython Notebooks using noWorkflow (Pimentel et al., 2017). PROV-O-Matic (Hoekstra, 2014) is another extension for earlier versions of IPython Notebooks to save the provenance traces to Linked Data file using PROV-O. In recent approaches, custom Jupyter kernels are developed to trace runtime user interactions and automatically manage the lineage of cell execution (Koop & Patel, 2017; Macke et al., 2021). However, some of these approaches do not capture the execution history of computational notebooks, require changes to the code by the user, and are limited to Python scripts. In our approach, the provenance tracking feature is integrated within a notebook, so there is no need for users to change the scripts and learn a new tool. We also make available the provenance information in an interoperable way.

There exists a gap in the current state-of-the-art systems as they do not interlink the data, the steps, and the results from both the computational and non-computational processes of a scientific experiment. We bridge this gap by developing a framework to capture the provenance, provide semantic integration of experimental data and support computational reproducibility. Hence, it is important to extend the current tools and at the same time, reuse their rich features to support the reproducibility and understandability of scientific experiments.

## Materials & Methods

### Requirement analysis

The prerequisite to developing an end-to-end provenance management platform arises from the requirements collected from interviews we conducted with scientists working in the Collaborative Research Center (CRC) ReceptorLight, as well as a workshop conducted to foster reproducible science (BEXIS2, 2017). These scientists come from different disciplines, including Biology, Computer Science, Ecology, and Chemistry. We also conducted an exploratory study to understand scientific experiments and the research practices of scientists related to reproducibility (Samuel & König-Ries, 2021). The detailed insights from these meetings and the survey helped us design, develop, and evaluate CAESAR. We developed the platform as part of the ReceptorLight project. Each module developed in CAESAR went through different stages like understanding requirements and use cases, designing the model, developing a prototype, testing and validating the prototype, and finally evaluating the work. As the end-users, several doctoral students and researchers from different disciplines were involved in each stage of the work. We used the feedback received from the domain researchers at each phase to improve the framework.

We describe here a summary of the insights of the interviews on the research practices of scientists. A scientific experiment consists of non-computational and computational data and steps. Computational tools like computers, software, scripts, *etc.*, generate computational data. Activities in the laboratory like preparing solutions, setting up the experimental execution environment, manual interviews, observations, *etc.*, are examples of non-computational activities. Measures taken to reproduce a non-computational step are different than those for a computational step. The reproducibility of a non-computational step depends on various factors like the availability of experiment materials (*e.g.*, animal cells or tissues) and instruments, the origin of the materials (*e.g.*, distributor of the reagents), human and machine errors, *etc.* Hence, non-computational steps need to be described in sufficient detail for their reproducibility (Kaiser, 2015).

The conventional way of recording the experiments in hand-written lab notebooks is still in use in biology and medicine. This creates a problem when researchers leave projects and join new projects. To understand the previous work conducted in a research project, all the information regarding the project, including previously conducted experiments along with the trials, analysis, and results, must be available to the new researchers. This information is also required when scientists are working on big collaborative projects. In their daily research work, a lot of data is generated and consumed through the computational and non-computational steps of an experiment. Different entities like devices, procedures, protocols, settings, computational tools, and execution environment attributes are involved in experiments. Several people play various roles in different steps and processes of an experiment. The outputs of some non-computational steps are used as inputs to the computational steps. Hence, an experiment must not only be linked to its results but also to different entities, people, activities, steps, and resources. Therefore, the complete path towards the results of an experiment must be shared and described in an interoperable manner to avoid conflicts in experimental outputs.

#### Design and development

We aim to design a provenance-based semantic framework for the end-to-end management of scientific experiments, including the computational and non-computational steps. To achieve our aim, we focused on the following modules: *provenance capture*, *representation*, *management*, *comparison*, and *visualization*. We used an iterative and layered approach in the design and development of CAESAR. We first investigated the existing frameworks that capture and store the experimental metadata and the data for the provenance capture module. We further narrowed down our search to imaging-based data management systems due to the extensive use of images and instruments in the experimental workflows in the ReceptorLight project. Based on our requirements, we selected OMERO as the underlying framework for developing CAESAR. Very active development community ensuring a continued effort to improve the system, a faster release cycle, a well-documented API to write own tools, and the ability to extend the web interface with plugins provided additional benefits to OMERO. However, they lack in providing the provenance support of experimental data, including the computational processes, and also lack in semantically representing the experiments. We designed and developed CAESAR by extending OMERO to capture the provenance of scientific experiments.

For the *provenance representation* module, we use semantic web technologies to describe the heterogeneous experimental data as machine-readable and link them with other datasets on the web. We develop the REPRODUCE-ME data model and ontology by extending existing web standards, PROV-O (Lebo et al., 2013) and P-Plan (Garijo & Gil, 2012). The REPRODUCE-ME Data Model is a generic data model for representing scientific experiments with their provenance information. An Experiment is considered as the central point of the REPRODUCE-ME data model. The model consists of eight components: Data, Agent, Activity, Plan, Step, Setting, Instrument, Material. We developed the ontology from the competency questions collected from the scientists in the requirement analysis phase (Samuel, 2019a). It is extended from PROV-O to represent all agents, activities, and entities involved in an experiment. It extends from P-Plan to represent the steps, the input and output variables, and the complete path taken from an input to an output of an experiment. Using the REPRODUCE-ME ontology, we can describe and semantically query the information for scientific experiments, input and output associated with an experiment, execution environmental attributes, experiment materials, steps, the execution order of steps and activities, agents involved and their roles, script/Jupyter Notebook executions, instruments, and their settings, *etc.* The ontology also consists of classes and properties, which describe the elements responsible for the image acquisition process in a microscope, from OME data model (OME, 2016).

For the *provenance management* module, we use PostgreSQL database and Ontology-based Data Access (OBDA) approach (Poggi et al., 2008). OBDA is an approach to access the various data sources using ontologies and mappings. The details of the structure of the underlying data sources are isolated from the users using a high-level global schema provided by ontologies. It helps to efficiently access a large amount of data from different sources and avoid replication of data that is already available in relational databases. It also provides many high-quality services to domain scientists without worrying about the underlying technologies. There are different widely-used applications involving large data sources that use OBDA  (Brüggemann et al., 2016; Kharlamov et al., 2017). As the image metadata in OMERO and the experimental data in CAESAR are already stored in the PostgreSQL database, we investigated the effective ways to represent scientific experiments’ provenance information without duplicating the data. Based on this, we selected to use the OBDA approach to represent this data semantically and at the same time avoid replication of data. To access the various databases in CAESAR, we used Ontop (Calvanese et al., 2017) for OBDA. We use the REPRODUCE-ME ontology to map the relational data in the OMERO and the ReceptorLight database using Ontop’s native mapping language. We used federation for the OMERO and ReceptorLight databases provided by the rdf4j SPARQL Endpoint (https://rdf4j.org/). We used the *Protege* plugin provided by Ontop to write the mappings. A virtual RDF graph is created in OBDA using the ontology with the mappings (Calvanese et al., 2017). SPARQL, the standard query language in the semantic web community, is used to query the provenance graph. We used the approach where the RDF graphs are kept virtual and queried only during query execution. The virtual approach helps avoid the materialization cost and provides the benefits of the matured relational database systems. However, there are some limitations in this approach using Ontop due to unsupported functions and data type.

To support computational reproducibility in CAESAR, we focused on providing the management of the provenance of the computational parts of an experiment. Computational notebooks, which have gained widespread attention because of their support for reproducible research, motivated us to look into this direction. These notebooks, which are extensively used and openly available, provide various features to run and share the code and results. We installed JupyterHub (https://jupyter.org/hub) in CAESAR to provide users access to computational environment and resources. JupyterHub provides a customizable and scalable way to serve Jupyter notebook for multiple users. In spite of the support for reproducible research, the provenance information of the execution of these notebooks was missing. To further support reproducibility in these notebooks, we developed ProvBook, an extension of Jupyter Notebooks, to capture the provenance information of their executions. We keep the design of ProvBook simple so that it can be used by researchers irrespective of their disciplines. We added the support to compare the differences in executions of the notebooks by different authors. We also extended the REPRODUCE-ME ontology to describe the computational experiments, including scripts and notebooks (Samuel & König-Ries, 2018a), which was missing in the current state of the art.

For the *provenance visualization* module, we focused on visualizing the complete path of an experiment by linking the non-computational and computational data and steps. Our two goals in designing the visualization component in CAESAR are providing users with a complete picture of an experiment and tracking its provenance. To do so, we integrated the REPRODUCE-ME ontology and ProvBook in CAESAR. We developed visualization modules to provide a complete story of an experiment starting from its design to publication. The visualization module, Project Dashboard, provides a complete overview of all the experiments conducted in a research project (Samuel et al., 2018). We later developed the ProvTrack module to track the provenance of individual scientific experiments. The underlying technologies are transparent to scientists based on these approaches. We followed a Model-View-Controller architecture pattern for the development of CAESAR. We implement the webclient in the Django-Python framework and the Dashboard in ReactJs. Java is used to implement the new services extended by OMERO.server. We use the D3 JavaScript library (D3.js, 2021) for the rendering of provenance graphs in the ProvTrack.

## Results

**Figure 1:**
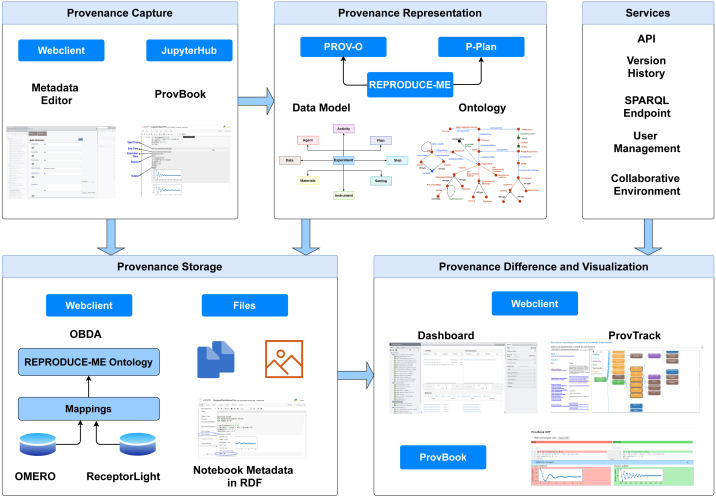
The architecture of CAESAR. The data management platform consists of modules for provenance capture, representation, storage, comparison, and visualization. It also includes several additional services including API access, and SPARQL endpoint.

We present CAESAR (**C**oll**A**borative **E**nvironment for **S**cientific **A**nalysis with **R**eproducibility), an end-to-end semantic-based provenance management platform. It is extended from OMERO (Allan et al., 2012). With the integration of the rich features provided by OMERO and our provenance-based extensions, CAESAR provides a platform to support scientists to describe, preserve and visualize their experimental data by linking the datasets with the experiments along with the execution environment and images. It provides extensive features, including the semantic representation of experimental data using the REPRODUCE-ME ontology and computational reproducibility support using ProvBook. Figure 1 depicts the architecture of CAESAR. We describe briefly the core modules of CAESAR required for the end-to-end provenance management.

### Provenance capture

This module provides a metadata editor with a rich set of features allowing the scientists to easily record all the data of the non-computational steps performed in their experiments and the protocols, the materials, *etc.* This metadata editor is a form-based provenance capture system. It provides the feature to document the experimental metadata and interlink with other experiment databases. An *Experiment* form is the key part of this system that documents all the information about an experiment. This data includes the temporal and spatial properties, the experiment’s research context, and other general settings used in the experiment. The materials and other resources used in an experiment are added as new templates and linked to the experiment. The templates are added as a service as well as a database table in CAESAR and are also available as API, thus allowing the remote clients to use them.

The user and group management provided by OMERO is adopted in CAESAR to manage users in groups and provides roles for these users. The restriction and modification of data are managed using the roles and permissions that are assigned to the users belonging to a group. The data is made available between the users in the same group in the same CAESAR server. Members of other groups can share the data based on the group’s permission level. A user can be assigned any of the role of *Administrator*, *Group Owner* or *Group Member*. An *Administrator* controls all the settings of the groups. A *Group Owner* has the right to add other members to the group. A *Group Member* is a standard user in the group. There are also various permission levels in the system. *Private* is the most restrictive permission level, thus providing the least collaboration level with other groups in the system. A private group owner can view and control the members and data of the members within a group; While a private group member can view and control only his/her data. The *Read-only* is an intermediate permission level. In addition to their group, the group owners can read and perform some annotations on members’ data from other groups. The group members don’t have permission to annotate the datasets from other groups, thus providing only viewing and reading possibilities. The *Read-annotate* permission level provides a more collaborative option where the group owners and members can view the other groups’ members as well as read and annotate their data. The *Read-write* permission level allows the group members to read and write data just like their own group.

CAESAR adopts this role and permission levels to control the access and modification of provenance information of experiments. A Principal Investigator (PI) can act as a group owner and students as group members in a *private* group. PIs can access students’ stored data and decide which data can be used to share with other collaborative groups. A *Read-only* group can serve as a public repository where the original data and results for the publications are stored. A *Read-annotate* group is suitable for collaborative teams to work together for a publication or research. Every group member is trusted and given equal rights to view and access the data in a *Read-write* group, thus providing a very collaborative environment. This user and group management paves the way for collaboration among teams in research groups and institutes before the publication is made available online.

This module also allows scientists to interlink the dependencies of many materials, samples, input files, measurement files, images, standard operating procedures, and steps to an experiment. Users can also attach files, scripts, publications, or other resources to any steps in an experiment form. They can annotate these resources as an input to a step or intermediate result of a step. Another feature of this module is to help the scientists to reuse resources rather than do it from scratch, thus enabling a collaborative environment among teams and avoiding replicating the experimental data. This is possible by sharing the descriptions of the experiments, standard operating procedures, and materials with the team members within the research group. Scientists can reference the descriptions of the resources in their experiments. Version management plays a key role in data provenance. In a collaborative environment, where the experimental data are shared among the team members, it is important to know the modifications made by the members of the system and track the history of the outcome of an experiment. This module provides version management of the experimental metadata by managing all the changes made in the description of the experimental data. The plugin allows users to view the version history and compare two different versions of an experiment description. The file management system in CAESAR stores all files and index them to the experiment, which is annotated as input data, measurement data, or other resources. The user can organize the input data, measurement data, or other resources in a hierarchical structure based on their experiments and measurements using this plugin.

CAESAR also provides a database of Standard Operating Procedures. These procedures in life-sciences provide a set of step-by-step instructions to carry out a complex routine. In this database, the users can store the protocols, procedures, scripts, or Jupyter Notebooks based on their experiments, which have multiple non-computational and computational steps. The users can also link these procedures to the step in an experiment where they were used. The plugin also provides users the facility to annotate the experimental data with terms from other ontologies like GO (Ashburner et al., 2000), CMPO (Jupp et al., 2016), *etc.* in addition to REPRODUCE-ME Ontology.

If a user is restricted to make modifications to other members’ data due to permission level, the plugin provides a feature called *Proposal* to allow users to propose changes or suggestions to the experiment. As a result, the experiment owner receives those suggestions as proposals. The user can either accept the proposal and add it to the current experimental data or reject and delete the proposal. The plugin provides autocompletion of data to fasten the process of documentation. For example, based on the CAS number of the chemical provided by the user, the molecular weight, mass, structural formulas are fetched from the CAS registry and populated in the Chemical database. The plugin also provides additional data from the external servers for other materials like Protein, Plasmid, and Vector. The plugin also autofills the data about the authors and other publication details based on the DOI/PubMedId of the publications. It also provides a virtual keyboard to aid the users in documenting descriptions with special characters, chemical formulas, or symbols.

### Provenance management and representation

We use a PostgreSQL database in OMERO as well as in CAESAR. The OMERO database consists of 145 tables, and the ReceptorLight database consists of 35 tables in total. We use the REPRODUCE-ME ontology to model and describe the experiments and their provenance in CAESAR. The database model and its schema consist of important classes which are based on the REPRODUCE-ME Data Model (Samuel, 2019a). For the data management of images, the main classes include Project, Dataset, Folder, Plate, Screen, Experiment, Experimenter, ExperimenterGroup, Instrument, Image, StructuredAnnotations, and ROI. Each class provides a rich set of features, including how they are used in an experiment. The *Experiment*, which is a subclass of *Plan* (Garijo & Gil, 2012), links all the provenance information of a scientific experiment together.

We use the REPRODUCE-ME ontology to map the relational data in the OMERO and the ReceptorLight database using Ontop. The provenance information of computational notebooks is also semantically represented and is combined with other experimental metadata, thus providing the context of the results. This helps the scientists and machines to understand the experiments along with their context. There are around 800 mappings to create the virtual RDF graph. All the mappings are publicly available. We refer the authors to the publication for the complete documentation of the REPRODUCE-ME ontology (Samuel, 2019b) and the database schema is available in the Supplementary file.

### Computational reproducibility

The introduction of computational notebooks, which allow scientists to share the code along with the documentation, is a step towards computational reproducibility. Scientists widely use Jupyter Notebooks to perform several tasks, including image processing and analysis. As the experimental data and images are contained in the CAESAR itself, another requirement is to provide a computational environment for scientists to include the scripts that analyze the data stored in the platform. To create a collaborative research environment for the scientists working with images and Jupyter Notebooks, JupyterHub is installed and integrated with CAESAR. This allows the scientists to directly access the images and datasets stored in CAESAR using the API and perform data analysis or processing on them using Jupyter Notebooks. The new images and datasets created in the Jupyter Notebooks can then be uploaded and linked to the original experiments to CAESAR using the APIs. To capture the provenance traces of the computational steps in CAESAR, we introduce ProvBook, an extension of Jupyter notebooks to provide provenance support (Samuel & König-Ries, 2018b). It is an easy-to-use framework for scientists and developers to efficiently capture, compare, and visualize the provenance data of different executions of a notebook over time. To capture the provenance of computational steps and support computational reproducibility, ProvBook is installed in JupyterHub and integrated with CAESAR. We briefly describe the modules provided by ProvBook.

#### Capture, management, and representation

This module captures and stores the provenance of the execution of Jupyter Notebooks cells over the course of time. A Jupyter Notebook, stored as a JSON file format, is a dictionary with the following keys: metadata and cells. The metadata is a dictionary that contains information about the notebook, its cells, and outputs. The cell contains information on all cells, including the source, the type of the cell, and its metadata. As Jupyter notebooks allow the addition of custom metadata to its content, the provenance information captured by the ProvBook is added to the metadata of each cell of the notebook in the JSON format. ProvBook captures the provenance information, including the start and end time of each execution, the total time it took to run the code cell, the source code, and the output obtained during that particular execution.

The execution time for a computational task was added as part of the provenance metadata in a Notebook since it is important to check the performance of the task. The start and end times also act as an indicator of the execution order of the cells. Users can execute cells in any order, so adding the start and end time helps them check when a particular cell was last executed. The users can change the parameters and source code in each cell until they arrive at their expected result. This helps the user to track the history of all the executions to see which parameters were changed and how the results were derived.

ProvBook also provides a module that converts the computational notebooks along with the provenance information of their executions and execution environment attributes into RDF. The REPRODUCE-ME ontology represents this provenance information. ProvBook allows the user to export the notebook in RDF as a turtle file either from the user interface of the notebook or using the command line. The users can share a notebook and its provenance in RDF and convert it back to a notebook. The reproducibility service provided by ProvBook converts the provenance graph back to a computational notebook along with its provenance. The Jupyter notebooks and the provenance information captured by ProvBook in RDF are then linked to the provenance of the experimental metadata in CAESAR.

### Comparison

To reproduce an experiment by another agent different from the original one and confirm the original experimenter’s results, it is necessary to get the provenance information of its input and steps along with the original results. ProvBook provides a feature that helps scientists compare the results of different executions of a Jupyter Notebook performed by the same or different agents.

ProvBook provides a provenance difference module to compare the different executions of each cell of a notebook, thus helping the users either to (1) repeat and confirm/refute their results or (2) reproduce and confirm/refute others results.

**Figure 2:**
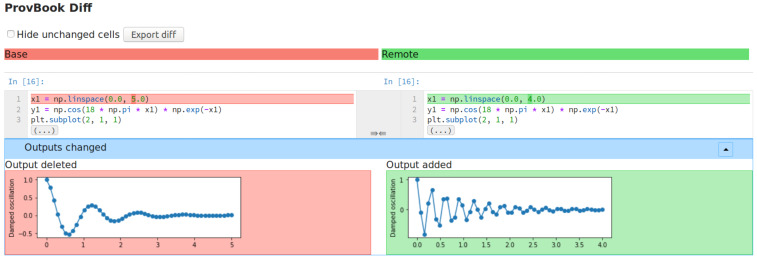
The difference between the input and output of two different execution of a code cell in ProvBook. Deleted elements are marked in red, newly added or created elements are marked in green.

ProvBook uses the start time of different executions collected to distinguish between the two executions. We provide a dropdown menu to select two executions based on their starting time. After the two executions are selected by the user, the difference in the input and the output of these executions are shown side by side. The users can select their own execution and compare the results with the original experimenter’s execution of the Jupyter Notebook. Figure 2 shows the differences between the source and output of two different code cell execution. ProvBook highlights any differences in the source or output for the user to distinguish the change. The provenance difference module is developed by extending the nbdime (Project Jupyter, 2021) library from the Project Jupyter. The nbdime tools provide the ability to compare notebooks and also a three-way merge of notebooks with auto-conflict resolution. ProvBook calls the API from the nbdime to see the difference between the provenance of two executions of a notebook code cell. Using nbdime, ProvBook provides diffing of notebooks based on the content and renders image-diffs correctly. This module in CAESAR helps scientists to compare the results of the executions of different users.

### Visualization

Efficient visualization is important to make meaningful interpretations of the data. The provenance information of each cell captured by ProvBook is visualized below every input cell. In this provenance area, a slider is provided so that the user can drag to view the history of the different executions of the cell. This area also provides the user with the ability to track the history and compare the current results with several previous results and see the difference that occurred. The user can visualize the provenance information of a selected cell or all cells by clicking on the respective buttons in the toolbar. The user can also clear the provenance information of a selected cell or all cells if required. This solution tries to address the problem of having larger provenance information than the original notebook data.

### Provenance visualization

We have described above the visualization of the provenance of cells in Jupyter Notebooks. In this section, we look at the visualization of overall scientific experiments. We present two modules for the visualization of the provenance information of scientific experiments captured, stored, and semantically represented in CAESAR: *Dashboard* and *ProvTrack*. The experimental data provided by scientists through the metadata editor, the metadata extracted from the images and instruments, and the details of the computational steps collected from ProvBook together are integrated, linked, and represented using the REPRODUCE-ME ontology. All this provenance data, stored as linked data, form the basis for the complete path of a scientific experiment and is visualized in CAESAR.

### Dashboard

This visualization module aggregates all the data related to an experiment and project in a single place. We provide users with two views: one at the project level and another at the experiment level. The Dashboard at the project level provides a unified view of a research project containing multiple experiments by different agents. When a user selects a project, the Project Dashboard is activated, while the Experiment Dashboard is activated when a dataset is selected.

The Dashboard is composed of several panels. Each panel provides a detailed view of a particular component of an experiment. The data inside a panel is displayed in tables. The panels are arranged in a way that they provide the story of an experiment. A detailed description of each panel is provided (Samuel et al., 2018). Users can also search and filter the data based on keywords inside a table in the panel.

### ProvTrack

**Figure 3:**
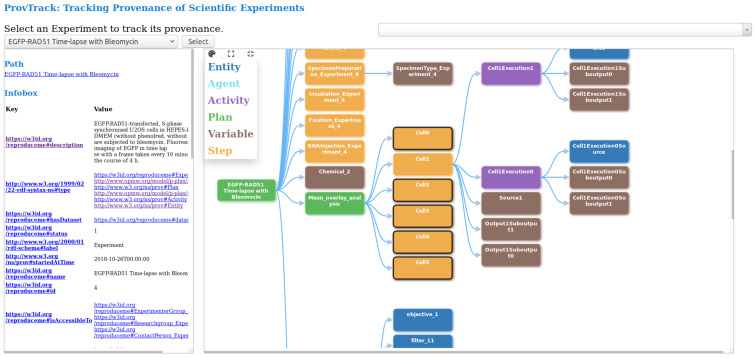
ProvTrack: tracking provenance of scientific experiments.

This visualization module provides users with an interactive way to track the provenance of experimental results. The provenance of experiments is provided using a node-link representation, thus, helping the user to backtrack the results. Users can drill-down each node to get more information and attributes. This module which is developed independently, is integrated into CAESAR. The provenance graph is based on the data model represented by REPRODUCE-ME ontology. We query the SPARQL endpoint to get the complete path of a scientific experiment. We make several SPARQL queries, and the results are combined to display this complete path and increase the system’s performance. Figure 3 shows the visualization of the provenance of an experiment using ProvTrack. CAESAR allows users to select an experiment to track its provenance.

The provenance graph is visualized in the right panel when the user selects an experiment. Each node in the provenance graph is colored based on its type like *prov:Entity*, *prov:Agent*, *prov:Activity*, *p-plan:Step*, *p-plan:Plan* and *p-plan:Variable*. The user can expand the provenance graph by opening up all nodes using the *Expand All* button next to the help menu. Using the *Collapse All* button, users can collapse the provenance graph to one node, which is the *Experiment*. ProvTrack shows the property relationship between two nodes when a user hovers on an edge. The help menu provides the user with the meaning of each color in the graph. The path from the user-selected node to the first node (Experiment) is highlighted to show the relationship of each node with the Experiment and also to see where the node is in the provenance graph.

ProvTrack also provides an *Infobox* of the selected node of an experiment. It displays the additional information about the selected node as a key-value pair. The keys in the Infobox are either the object or data properties of the REPRODUCE-ME ontology that is associated with the node that the user has clicked. Links are provided to the keys to get their definitions from the web. It also displays the path of the selected node from the Experiment node on top of the left panel. The Search panel allows the users to search for any entities in the graph defined by the REPRODUCE-ME data model. It provides a dropdown to search not only the nodes but also the edges. This comes handy when the provenance graph is very large.

### Evaluation

We evaluate different aspects of our work based on our main research question: How can we capture, represent, manage and visualize a complete path taken by a scientist in an experiment, including the computational and non-computational steps to derive a path towards experimental results? As the main research question has a broad scope and it is challenging to evaluate every part within the limited time and resources, we break the main research questions into smaller parts. We divide the evaluation into three parts based on the smaller questions and discuss their results separately. In the first part, we address the question of capturing and representing the complete path of a scientific experiment which includes both computational and non-computational steps. For this, we use the non-computational data captured in CAESAR and the computational data captured using ProvBook. The role of the REPRODUCE-ME ontology in semantically representing the complete path of a scientific experiment is evaluated using the knowledge base in CAESAR using the competency question-based evaluation. In the second part, we address the question of supporting reproducibility by capturing and representing the provenance of computational experiments. For this, we address the role of ProvBook in terms of reproducibility, performance, and scalability. We focused on evaluating ProvBook as a stand-alone tool and also with the integration with CAESAR. In the third part, we address the question of representing and visualizing the complete path of scientific experiments to the users of CAESAR. For this, we performed an evaluation by conducting a user-based study to get general impression of the tool and use this as a feedback to improve the tool. Scientists from within and outside the project were involved in all the three parts of our evaluation as system’s users as well as participants.

#### Competency question-based evaluation

Brank, Grobelnik & Mladenic (2005) point out different methods of evaluating ontologies. In application-based evaluation, the ontology under evaluation is used in an application/system to produce good results on a given task. Answering the competency questions over a knowledge base is one of the approaches to testing ontologies (Noy, McGuinness et al., 2001). Here, we applied the ontology in an application system and answered the competency questions over a knowledge base. Hence, we evaluated CAESAR with the REPRODUCE-ME ontology using competency questions collected from different scientists in our requirement analysis phase. We used the REPRODUCE-ME ontology to answer the competency questions using the scientific experiments documented in CAESAR for its evaluation. We did the evaluation on a server (installed with CentOS Linux 7 and with x86-64 architecture) hosted at the University Hospital Jena. To address the first part of the main research question, scientists from B1 and A4 projects of ReceptorLight documented experiments using confocal patch-clamp fluorometry (cPCF), Förster Resonance Energy Transfer (FRET), PhotoActivated Localization Microscopy (PALM) and direct Stochastic Optical Reconstruction Microscopy (dSTORM) as part of their daily work. In 23 projects, a total of 44 experiments were recorded and uploaded with 373 microscopy images generated from different instruments with various settings using either the desktop client or webclient of CAESAR (Accessed 21 April 2019). We also used the Image Data Repository (IDR) datasets (IDR, 2021) with around 35 imaging experiments (Williams et al., 2017) for our evaluation to ensure that the REPRODUCE-ME ontology can be used to describe other types of experiments as well. The description of the scientific experiments, along with the steps, experiment materials, settings, and standard operating procedures, using the REPRODUCE-ME ontology is available in CAESAR to its users and the evaluation participants. The scientific experiments that used Jupyter Notebooks are linked with the provenance of these notebooks captured using ProvBook in CAESAR. This information described using the REPRODUCE-ME ontology is also available in CAESAR for the participants (Listings 2). We created a knowledge base of different types of experiments from these two sources. The competency questions, which were translated into SPARQL queries by computer scientists, were executed on our knowledge base, consisting of linked data in CAESAR. The domain experts evaluated the correctness of the answers to these competency questions. We present here one competency question with the corresponding SPARQL query and part of the results obtained on running it against the knowledge base. The result of each query is a long list of values, hence, we show only the first few rows from them. This query is responsible for getting the complete path for an experiment.


 
 
Listing 1: What is the complete path taken by a scientist for an experiment?_ 
SELECT   DISTINCT   ∗ WHERE 
{ 
   ?experiment  a  repr:Experiment  ; 
           prov:wasAttributedTo  ?agent  ;   repr:hasDataset  ? dataset   ; 
           prov:generatedAtTime  ?generatedAtTime  . 
       ?agent  repr:hasRole  ? role   . 
       ? dataset  prov:hadMember  ?image  . 
       ?instrument  p−plan:correspondsToVariable  ?image  ; 
           repr:hasPart  ? instrument _part   . 
       ? instrument _part   repr:hasSetting  ? setting   . 
       ?plan  p−plan:isSubPlanOfPlan  ?experiment  . 
       ? variable  p−plan:isVariableOfPlan  ?plan  . 
       ? step  p−plan:isStepOfPlan  ?experiment  . 
       OPTIONAL   { ? step  p−plan:isPrecededBy  ? previousStep  } . 
       { 
     ?Input  p−plan:isInputVarOf  ? step   ;   rdf:type  ?InputType  . 
            OPTIONAL   { ?Input  repr:name  ?InputName  } . 
       } 
   UNION   { 
     ?Output  p−plan:isOutputVarOf  ? step   ; 
                rdf:type  ?OutputType  . 
            OPTIONAL   { ?Output  repr:name  ?OutputName  } . 
            OPTIONAL   { ?Output  repr:isAvailableAt  ?outputUrl  } . 
            OPTIONAL   { ?Output  repr:reference  ?OutputReference  . 
                   ?OutputReference  rdf:value  ?OutputReferenceValue 
            } 
   } 
}________________________________________________________________    


**Figure 4:**
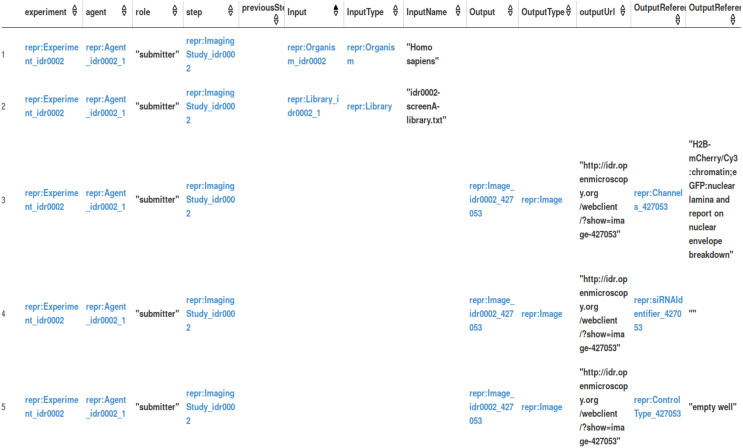
A part of results for the competency question.

Figure 4 shows the part of the result for a particular experiment called ‘Focused mitotic chromosome condensation screen using HeLa cells’. Here, we queried the experiment with its associated agents and their role, the plans and steps involved, the input and output of each step, the order of steps, and the instruments and their setting. We see that all these elements are linked now to the computational and non-computational steps to describe the complete path. We can further expand this query by asking for additional information like the materials, publications, external resources, methods, *etc.*, used in each step of an experiment. It is possible to query for all the elements mentioned in the REPRODUCE-ME Data Model.

The domain experts reviewed, manually compared, and evaluated the correctness of the results from the queries using the Dashboard. Since the domain experts do not possess the knowledge of SPARQL, they did this verification process with the help of the Dashboard. This helped them get a complete view of the provenance of scientific experiments. In this verification process, the domain experts observed that the query returned null for certain experiments that did not provide the complete data for some elements. So the computer scientists from the project tweaked the query to include the OPTIONAL keyword to get the results from the query. Even after tweaking the results, the results from these competency questions were not complete. Missing data is especially seen in the non-computational part of an experiment than its computational part. This shows that the metadata entered by the scientists in CAESAR is not complete and requires continuous manual annotation. Another thing that we noticed during the evaluation is that the results are spread across several rows in the table. In the Dashboard, when we show these results, the filter option provided in the table helps the user to search for particular columns. The domain experts manually compared the results of SPARQL queries using Dashboard and ProvTrack and evaluated their correctness (Samuel et al., 2018). Each competency question addressed the different elements of the REPRODUCE-ME Data Model. The competency questions, the RDF data used for the evaluation, the SPARQL queries, and the answers to these queries are publicly available (Samuel, 2021). The data provides ten competency questions which are translated to SPARQL queries. The answers to these SPARQL queries provide information on the steps, plans, methods, standard operating procedures, instruments and their settings, materials used in each step, agents directly and indirectly involved, and the temporal parameters of an experiment. To summarize, the domain scientists found the answers to the competency questions satisfactory with the additional help of computer scientists tweaking the SPARQL queries. However, the answers to the competency questions showed the missing provenance information, which needs user input in CAESAR.

### Data and user-based evaluation of ProvBook

In this section, we address how ProvBook supports computational reproducibility as a stand-alone tool and also with the integration with CAESAR. To address the second part of the main research question, we evaluate the role of ProvBook in supporting computational reproducibility using Jupyter Notebooks. We did the evaluation taking into consideration different use cases and factors. They are:

1. Repeatability: The computational experiment is repeated in the same environment by the same experimenter. We performed this to confirm the final results from the previous executions.
2. Reproducibility: The computational experiment is reproduced in a different environment by a different experimenter. In this case, we compare the results of the Jupyter notebook in the original environment with the results from a different experimenter executed in a different environment.
3. The input, output, execution time and the order in two different executions of a notebook.
4. Provenance difference of the results of a notebook.
5. Performance of ProvBook with respect to time and space.
6. The environmental attributes in the execution of a notebook.
7. Complete path taken by a computational experiment with the sequence of steps in the execution of a notebook with input parameters and intermediate results in each step required to generate the final output.

We used an example Jupyter Notebook which uses a face recognition example where eigenface and SVM algorithms from scikit-learn (https://scikit-learn.org/0.16/datasets/labeled_faces.html) are applied. This script provides a computational experiment to extract information from an image dataset that is publicly available using machine learning techniques. We used this example script to show the different use cases of our evaluation and how ProvBook handles different output formats like image, text, *etc.* These formats are also important for the users of CAESAR. We use *Original Author* to refer to the author who is the first author of the notebook and *User 1* and *User 2* to the authors who used the original notebook to reproduce results. We first saved the notebook without any outputs. Later, two different users executed this notebook in three different environments (Ubuntu 18.10 with Python 3, Ubuntu 18.04 with Python 2 and 3, Fedora with Python 3). Both users used ProvBook in their Jupyter Notebook environment. The first run of the eigenfaces Jupyter Notebook gave *ModuleNotFoundError* for *User 1*. *User 1* attempted several runs to solve the issue. For *User 1* installing the missing scikit-learn module still did not solve the issue. The problem occurred because of the version change of the scikit-learn module. The original Jupyter Notebook used 0.16 version of scikit-learn. While *User 1* used 0.20.0 version, *User 2* used 0.20.3. The classes and functions from the cross_validation, grid_search, and learning_curve modules were placed into a new model_selection module starting from Scikit-learn 0.18. *User 1* made several other changes in the script which used these functions. *User 1* also made the necessary changes to work for the new versions of the scikit-learn module. We provided this changed notebook along with the provenance information captured by ProvBook in *User 1* notebook environment to *User 2*. For *User 2*, only the first run gave *ModuleNotFoundError* error. *User 2* resolved this issue by installing the scikit-learn module. *User 2* did not have to change scripts, as the *User 2* could see the provenance history of the executions from the original author, *User 1* and his own execution. Using ProvBook, *User 1* could track the changes and compare with the original script, while *User 2* could compare the changes with the executions from the original author, *User 1* and his own execution. Since this example is based on a small number of users, an extensive user-based evaluation is required to conclude the role of ProvBook for the increased performance in these use cases. However, we expect that ProvBook play a role in helping users in such use cases to track the provenance of experiments.

We also performed tests to see the input, output, and run time in different executions in different environments. The files in the Supplementary information provides the information on this evaluation by showing the difference in the execution time of the same cell in a notebook in different execution environments. One of the cells in the evaluation notebook downloads a set of preprocessed images from Labeled Faces in the World (LFW) (http://vis-www.cs.umass.edu/lfw/) which contains the training data for face recognition study. The execution of this cell took around 41.3ms in the first environment (Ubuntu 18.10), 2 min 35s in the second environment (Ubuntu 18.04), and 3 min 55s in the third environment (Fedora). The different execution environments play a role in computational experiments which is shown with the help of ProvBook. We also show how one change in a previous cell of the notebook resulted in a difference in the intermediate result in two different executions by two different users in two different environment. We evaluated the provenance capture and difference module in ProvBook with different output types, including images. The results show that ProvBook can handle different output types, which Jupyter Notebooks support. We also evaluated the performance of ProvBook with respect to space and time. The difference in the run time of each cell with and without ProvBook was negligible. Concerning space, the size of the Jupyter Notebook with provenance information of several executions was more than the original notebook. This is stated in Chapman, Jagadish & Ramanan (2008) that the size of the provenance information can grow more than the actual data. In the following scenario, we evaluated the semantic representation of the provenance of computational notebooks by integrating ProvBook with CAESAR. Listings 2 shows the SPARQL query of the complete path for a computational notebook with input parameters and intermediate results in each step required to generate the final output. It also queries the sequence of steps in its execution.

 
 
     Listing 2: Complete path for a computational notebook experiment_______ 
SELECT   DISTINCT   ∗ WHERE 
{ 
   ? step  p−plan:isStepOfPlan  ?notebook  . 
       ?notebook  a  repr:Notebook  . 
       ? execution  p−plan:correspondsToStep  ? step   ; 
           repr:executionTime  ?executionTime  . 
       ? step  p−plan:hasInputVar  ?inputVar  ; 
           p−plan:hasOutputVar  ?outputVar  ; 
           p−plan:isPrecededBy  ? previousStep   . 
}________________________________________________________________    

We can expand this query to get information on the experiment in CAESAR which uses a notebook. The result of this query, which is available in the Supplementary file, shows the steps, the execution of each step with their total run time, the input and output data of each run, and the order of execution of steps of a notebook.

### User-based evaluation of CAESAR

This evaluation addresses a smaller component of the third part of the main research question. Here, we focus on the visualization of the complete path of scientific experiments to the users of CAESAR. We conducted a user-based study to get the general impression of the tool and used this as feedback to improve the tool. This study aimed to evaluate the usefulness of CAESAR and its different modules, particularly the visualization module. We invited seven participants for the survey, of which six participants responded to the questions. The evaluation participants were the scientists of the ReceptorLight project who use CAESAR in their daily work. In addition to them, other biology students, who closely work with microscopy images and are not part of the ReceptorLight project, participated in this evaluation. We provided an introduction of the tool to all the participants and provided a test system to explore all the features of CAESAR. The scientists from the ReceptorLight project were given training on CAESAR and its workflow on documenting experimental data. Apart from the internal meetings, we provided the training from 2016-2018 (17.06.2016, 19.07.2016, 07.06.2017, 09.04.2018, and 16.06.2018). We asked scientists to upload their experimental data to CAESAR as part of this training. At the evaluation time, we provided the participants with the system with real-life scientific experiment data as mentioned in the competency question evaluation subsection. The participants were given the system to explore all the features of CAESAR. As our goal of this user survey was to get feedback from the daily users and new users and improve upon their feedback, we let the participants answer the relevant questions. As a result, the user survey was not anonymous, and none of the questions in the user survey was mandatory. However, only 1 participant who was not part of the ReceptorLight project did not answer all the questions. The questionnaire and the responses are available in the Supplementary File.

In the first section of the study, we asked how the features in CAESAR help improve their daily research work. All the participants either strongly agreed or agreed that CAESAR enables them to organize their experimental data efficiently, preserve data for the newcomers, search all the data, provide a collaborative environment and link the experimental data with results. 83% of the participants either strongly agreed or agreed that it helps to visualize all the experimental data and results effectively, while 17% disagreed on that. In the next section, we asked about the perceived usefulness of CAESAR. A total of 60% of the users consider CAESAR user-friendly, while 40% had a neutral response. A total of 40% of the participants agreed that CAESAR is easy to learn to use, and 60% had a neutral response. The participants provided additional comments to this response that CAESAR offers many features, and they found it a little difficult to follow. However, all the participants strongly agreed or agreed that CAESAR is useful for scientific data management and provides a collaborative environment among teams.

In the last section, we evaluated each feature provided by CAESAR by focusing on the important visualization modules. Here, we showed a real-life scientific experiment with Dashboard and ProvTrack views. We asked the participant to explore the various information, including the different steps and materials used in the experiment. Based on their experience, we asked the participants about the likeability of the different modules. ProvTrack was strongly liked or liked by all the participants. For the Dashboard, 80% of them either strongly liked or liked, while 20% had a neutral response. A total of 60% of the users strongly liked or liked ProvBook, while the other 40% had a neutral response. The reason for the neutral response was that they were new to scripting. We also asked to provide the overall feedback of CAESAR along with its positive aspects and the things to improve. We obtained three responses to this question which are available in the Supplementary File.

## Discussion

Provenance plays a key role in supporting the reproducibility of results, which is an important concern in data-intensive science. Through CAESAR, we aimed to provide a data management platform for capturing, semantically representing, comparing, and visualizing the provenance of scientific experiments, including their computational and non-computational aspects. CAESAR is used and deployed in the CRC ReceptorLight project, where scientists work together to understand the function of membrane receptors and develop high-end light microscopy techniques. In the competency question-based evaluation, we focused on answering the questions using the experimental provenance data provided by scientists from the research projects, which was then managed and semantically described in CAESAR. Answering the competency questions using SPARQL queries shows that some experiments documented in CAESAR had missing provenance data on some of the elements of the REPRODUCE-ME Data Model like time, settings, *etc.* We see that CAESAR requires continuous user involvement and interaction in documenting non-computational parts of an experiment. Reproducing an experiment is currently not feasible unless every step in CAESAR is machine-controlled. In addition to that, the output of the query for finding the complete path of the scientific experiment results in many rows in the table. Therefore, the response time could exceed the normal query response time and result in server error from the SPARQL endpoint in some cases where the experiment has various inputs and outputs with several executions. Currently, scientists from the life sciences do not have the knowledge of Semantic Web technologies and are not familiar with writing their SPARQL queries. Hence, we did not perform any user study on writing SPARQL queries to answer competency questions. However, scientists must be able to see the answers to these competency questions and explore the complete path of a scientific experiment. To overcome this issue, we split the queries and combined their results in ProvTrack. The visualization modules, Dashboard, and ProvTrack, which use SPARQL and linked data in the background, visualize the provenance graph of each scientific experiment. ProvTrack groups the entities, agents, activities, steps, and plans to help users visualize the complete path of an experiment. In the data and user-based evaluation, we see the role of ProvBook as a stand-alone tool to capture the provenance history of computational experiments described using Jupyter Notebooks.

We see that each item added in the provenance information in Jupyter Notebooks, like the input, output, starting and ending time, helps users track the provenance of results even in different execution environments. The Jupyter Notebooks shared along with the provenance information of their executions helps users to compare the original intermediate and final results with the results from the new trials executed in the same or different environment. Through ProvBook, the intermediate and negative results and the input and the output from different trials are not lost. The execution environmental attributes of the computational experiments along with their results, help to understand their complete path. We also see that we could describe the relationship between the results, the execution environment, and the executions that generated the results of a computational experiment in an interoperable way using the REPRODUCE-ME ontology. The knowledge capture of computational experiments using notebooks and scripts is ongoing research, and many research questions are yet to be explored. ProvBook currently does not extract semantic information from the cells. This includes information like the libraries used, variables and functions defined, input parameters and output of a function, *etc.* In CAESAR, we currently link a whole cell as a step of a notebook which is linked to an experiment. Hence, the fine provenance information of a computational experiment is currently missing and thus not linked to an experiment to get the complete path of a scientific experiment.

The user-based evaluation of CAESAR aimed to see how the users find CAESAR useful concerning the features it provides. We targeted both the regular users and the new users to the system. As we had a small group of participants, we could not make general conclusions from the study. However, the study participant either agreed or liked its features. The survey results in Samuel & König-Ries (2021) had shown that newcomers face difficulty in finding, accessing, and reusing data in a team. We see an agreement among the participants that CAESAR helps preserve data for the newcomers to understand the ongoing work in the team. This understanding of the ongoing work in the team comes from the linking of experimental data and results. The results from the study show that among the two visualization modules, ProvTrack was preferred over Dashboard by scientists. Even though both serve different purposes (Dashboard for an overall view of the experiments conducted in a Project and the ProvTrack for backtracking the results of one experiment), the users preferred the provenance graph to be visualized with detailed information on clicking. The survey shows that the visualization of the experimental data and results using ProvTrack supported by the REPRODUCE-ME ontology helps the scientists without worrying about the underlying technologies. All the participants either strongly agreed or agreed that CAESAR enables them to organize their experimental data efficiently, preserve data for the newcomers, search all the data, provide a collaborative environment and link the experimental data with results. However, a visualization evaluation is required to properly test the Dashboard and ProvTrack views, which will help us to determine the usability of visualizing the complete path of a scientific experiment.

The limitation of our evaluation is the small number of user participation. Hence, we cannot make any statistical conclusion on the system’s usefulness. However, CAESAR is planned to be used and extended for another large research project, Microverse (https://microverse-cluster.de), which will allow for a more scalable user evaluation. One part of the provenance capture module depends on the scientists to document their experimental data. Even though the metadata from the images captures the execution environment and the devices’ settings, the need for human annotations to the experimental datasets is significant. Besides this limitation, the mappings for the ontology-based data access required some manual curation. This can affect when the database is extended for other experiment types.

## Conclusions

In this article, we presented CAESAR. It provides a collaborative framework for the management of scientific experiments, including the computational and non-computational steps. The provenance of the scientific experiments is captured and semantically represented using the REPRODUCE-ME ontology. ProvBook helps the user capture, represent, manage, visualize and compare the provenance of different executions of computational notebooks. CAESAR links the computational data and steps to the non-computational data and steps to represent the complete path of the experimental workflow. The visualization modules of CAESAR provides users to view the complete path and backtrack the provenance of results. We applied our contributions together in the ReceptorLight project to support the end-to-end provenance management from the beginning of an experiment to its end. There are several possibilities to extend and improve CAESAR. We expect this approach to be extended to different types of experiments in diverse scientific disciplines. Reproducibility of non-computational parts of an experiment is our future line of work. We can reduce the query time for the SPARQL queries in the project dashboard and ProvTrack by taking several performance measures. CAESAR could be extended to serve as a public data repository providing DOIs to the experimental data and provenance information. This would help the scientific community to track the complete path of the provenance of the results described in the scientific publications. Currently, CAESAR requires continuous user involvement and interaction, especially through different non-computational steps of an experiment. The integration of persistent identifiers for physical samples and materials into scientific data management can lower the effort of user involvement. However, at this stage, reproducibility is not a one-button solution where reproducing an experiment is not feasible unless every step is machine-controlled.

## Supplemental Information

10.7717/peerj-cs.921/supp-1 Supplemental Information 1 The Project Dashboard in CAESAR for the complete overview of experiments conducted in a project The projects, datasets, and experiments and the provenance information are displayed in tables in each panel.

10.7717/peerj-cs.921/supp-2 Supplemental Information 2 User Evaluation Figures of ProvBook Each figure shows the ProvBook in different scenarios. It contains the provenance difference in different runs and execution environment.

10.7717/peerj-cs.921/supp-3 Supplemental Information 3 The user evaluation questionnaire used for CAESAR Evaluation The purpose of this questionnaire was to see how the users find CAESAR useful with respect to the features it provides.

10.7717/peerj-cs.921/supp-4 Supplemental Information 4 The user responses of CAESAR evaluation along with the questionnaire The purpose of this study was to see how the users find CAESAR useful with respect to the features it provides.

10.7717/peerj-cs.921/supp-5 Supplemental Information 5 CAESAR User Manual The document provides the supplementary information on the provenance visualization and representation modules. The panels of the Dashboard are explained in detail. The main classes of CAESAR database is provided.

## Additional Information and Declarations

### Data Availability

The following information was supplied regarding data availability: The underlying code of CAESAR is available at GitHub: https://github.com/CaesarReceptorLight. The current production server is hosted at the Jena Hospital, and because of privacy and confidentiality reasons, it is currently not made publicly available. Therefore, the data used in the project ReceptorLight for evaluation is also not publicly available. The REPRODUCE-ME ontology is available at GitHub: https://github.com/Sheeba-Samuel/REPRODUCE-ME. The code for ProvBook is available at GitHub: https://github.com/Sheeba-Samuel/ProvBook. The resources used for the evaluation of CAESAR, ProvBook, and REPRODUCE-ME ontology are available at GitHub: https://github.com/Sheeba-Samuel/CAESAREvaluation
The questionnaire and the response for CAESAR User evaluation are available at GitHub and as Supplementary Files.
