# Supplementary material for: A collaborative semantic-based provenance management platform for reproducibility
Source: PeerJ Comput Sci. 2022 Mar 10;8:e921. doi: 10.7717/peerj-cs.921 (PMC9044346; doi:10.7717/peerj-cs.921)
Supplement: Supplemental Information 1 — The projects, datasets, and experiments and the provenance information are displayed in tables in each panel. [file peerj-cs-08-921-s001.pdf]

ReceptorLight_B1

Explore | Tags | Shares

- All Members
  - Binding on modulatory CNG subunits 14
    - cAMP/cGMP efficiency on A1
    - cAMP/cGMP efficiency on A1:B1a
    - ...e-binding A1-617-GFP:B1a via FRET
    - ...ding A1-617-GFP:B1adeltaB via FRET
    - dose-binding A1:B1a-1204-GFP
    - dose-binding A1:B1a-1204-GFP
    - dose-binding A1:B1adeltaB-1204-GFP
    - ...-response A1-617-GFP:B1a + cGMP
    - dose-response A1:B1a + cGMP
    - dose-response A1:B1a + fcGMP
    - dose-response A1:B1adeltaB
    - fcGMP affinity to CNGA1:B1a
    - ...ane integration of A1:B1a-1204-GFP
    - ...brane integration of B1a-1204-GFP
  - Binding via FRET 9
    - dose-binding A1-617-GFP
    - dose-response A1-617-GFP
    - dose-responses A1
    - fcGMP affinity to CNGA1
    - fcGMP affinity to CNGA1-617-GFP
    - fcGMP efficiency
    - FRET specificity to ligand binding
    - GFP bleaching
    - P1fcGMP washout
  - Example Data 2
    - Staining cellular compartments
    - Terminal Ganglion P. americana
  - Late HR Dynamics 8
    - ...ion of EGFP-RAD51 and Cherry-RAD54
    - EGFP-RAD51 time lapse
    - EGFP-RAD51/mCherry-RAD54

ProjectDashbc

The Plot

Experiment	Name	AgentName	AgentRole	ger
s://w3id.org/repro	Staining cellular com		Research Group	2019-
s://w3id.org/repro	Staining cellular com		Contact Person	2019-
s://w3id.org/repro	Staining cellular com	Bioimaging Practical	Project	2019-

Previous | Page 1 of 1 | 10 rows | Next

The Characters

Experiment	Agent	AgentName	Role
https://w3id.org/repro	https://w3id.org/repro	Bioimaging Practical	Project

Previous | Page 1 of 1 | 10 rows | Next

Materials

Vector | Plasmid | Protein | Chemical | Solution | DNA | RNA | Restriction Enzyme | Fluorescent Protein | Oligonucleotide

material	Name	usedin	usedinstep
https://w3id.org/reproduceme#Vector_1	pEGFP-C1	https://w3id.org/reproduceme#Vector_3	
https://w3id.org/reproduceme#Vector_1	pEGFP-C1	https://w3id.org/reproduceme#Vector_4	
https://w3id.org/reproduceme#Vector_1	pEGFP-C1	https://w3id.org/reproduceme#Plasmid_1	
https://w3id.org/reproduceme#Vector_1	pEGFP-C1	https://w3id.org/reproduceme#Plasmid_2	

General | Acquisition | Preview | Explorer | Experiment

Example Data

Project ID: 104
Owner: Tom Kache

Show al

Project Details

Add Description

Creation Date: 2019-03-01 21:24:11

- Tags 0
- Key-Value Pairs 0
- Attachments 0
- Comments 0
- Ratings 0
- Others 0
