# Supplementary figures and images for: A collaborative semantic-based provenance management platform for reproducibility
Source: PeerJ Comput Sci. 2022 Mar 10;8:e921. doi: 10.7717/peerj-cs.921 (PMC9044346; doi:10.7717/peerj-cs.921)

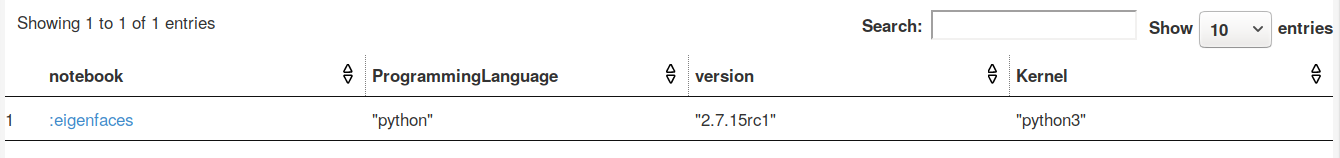

Supplement: Supplemental Information 2 — Each figure shows the ProvBook in different scenarios. It contains the provenance difference in different runs and execution environment. [file peerj-cs-08-921-s002.zip › Evaluation/SupplementaryFile/Environment.png]

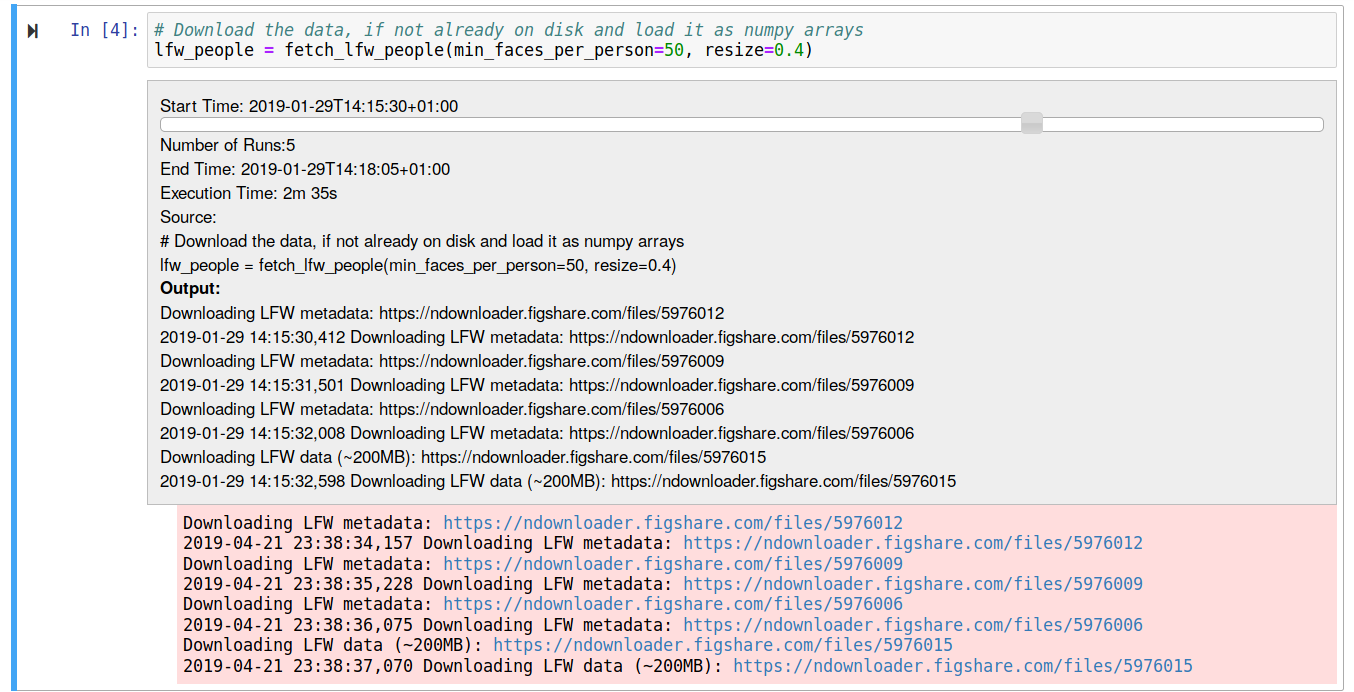

Supplement: Supplemental Information 2 — Each figure shows the ProvBook in different scenarios. It contains the provenance difference in different runs and execution environment. [file peerj-cs-08-921-s002.zip › Evaluation/SupplementaryFile/ExecutionEnvironment_Fedora.png]

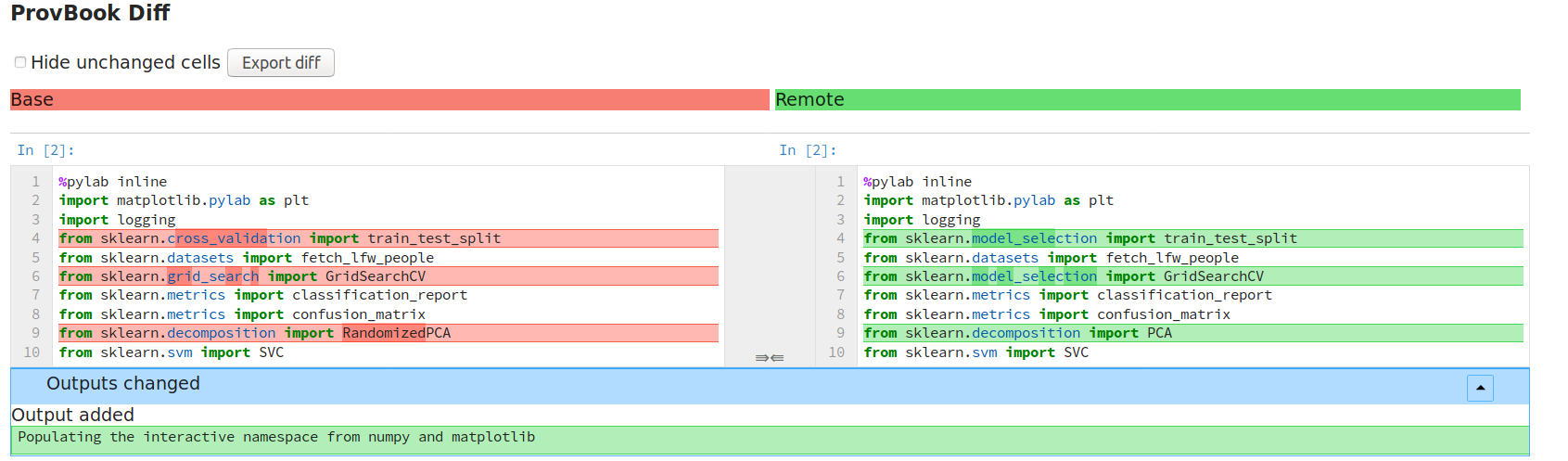

Supplement: Supplemental Information 2 — Each figure shows the ProvBook in different scenarios. It contains the provenance difference in different runs and execution environment. [file peerj-cs-08-921-s002.zip › Evaluation/SupplementaryFile/User1_FirstCellFifthRun.png]

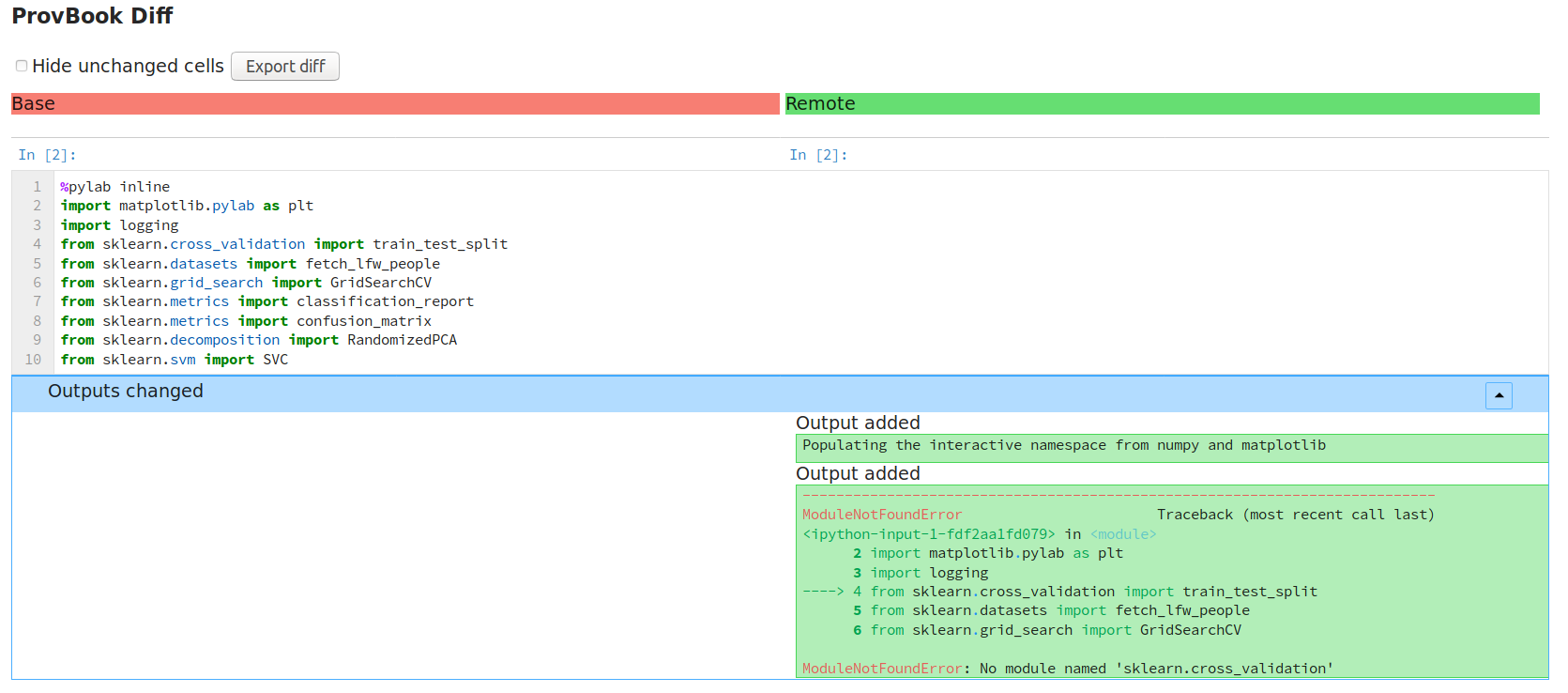

Supplement: Supplemental Information 2 — Each figure shows the ProvBook in different scenarios. It contains the provenance difference in different runs and execution environment. [file peerj-cs-08-921-s002.zip › Evaluation/SupplementaryFile/User1_FirstCellFirstRun.png]

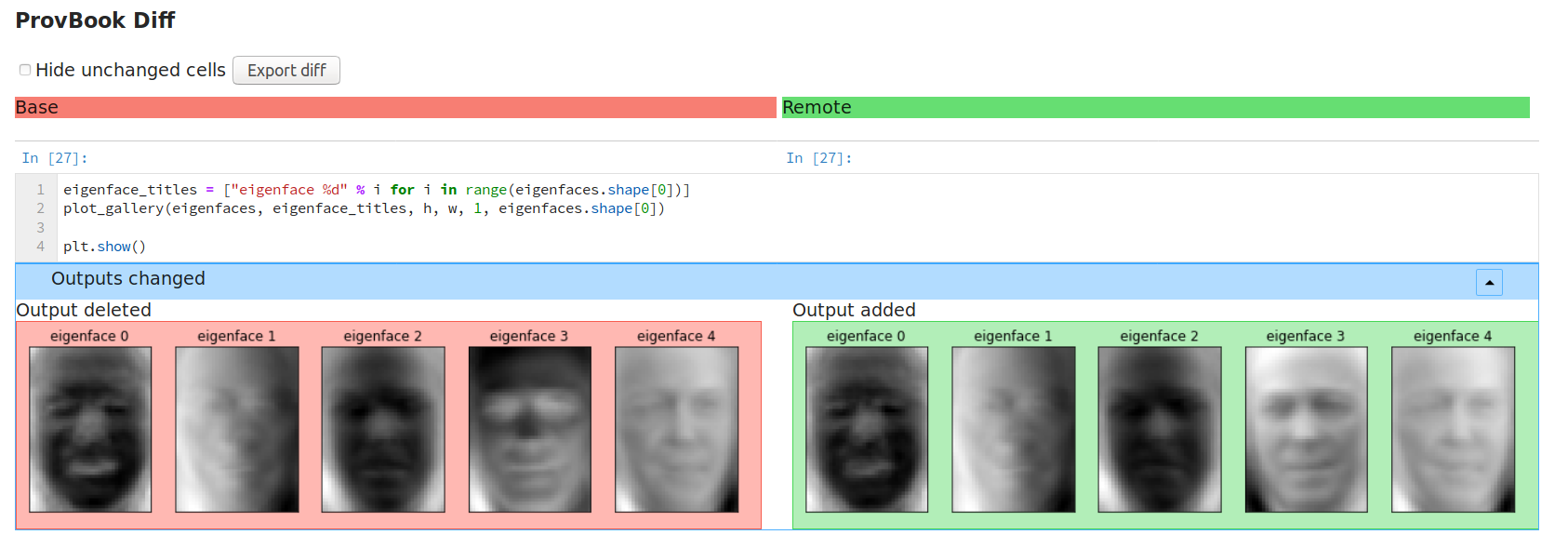

Supplement: Supplemental Information 2 — Each figure shows the ProvBook in different scenarios. It contains the provenance difference in different runs and execution environment. [file peerj-cs-08-921-s002.zip › Evaluation/SupplementaryFile/Difference_Image.png]

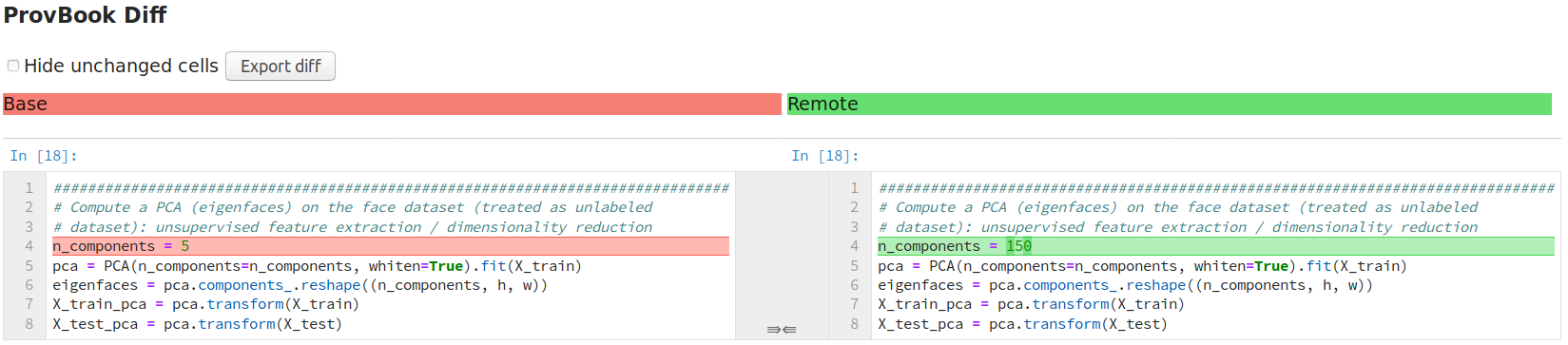

Supplement: Supplemental Information 2 — Each figure shows the ProvBook in different scenarios. It contains the provenance difference in different runs and execution environment. [file peerj-cs-08-921-s002.zip › Evaluation/SupplementaryFile/Difference_Input.png]

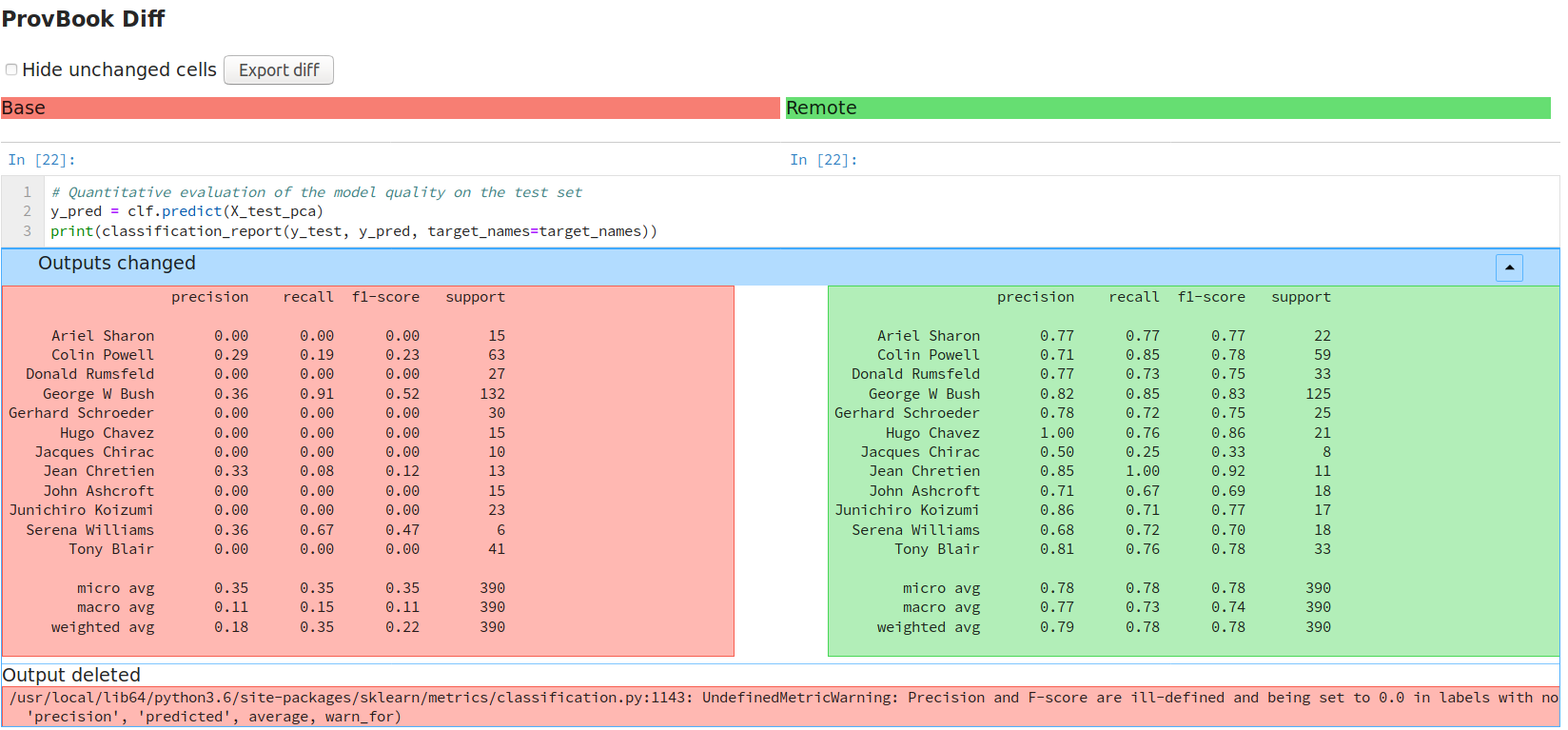

Supplement: Supplemental Information 2 — Each figure shows the ProvBook in different scenarios. It contains the provenance difference in different runs and execution environment. [file peerj-cs-08-921-s002.zip › Evaluation/SupplementaryFile/Difference_Output.png]

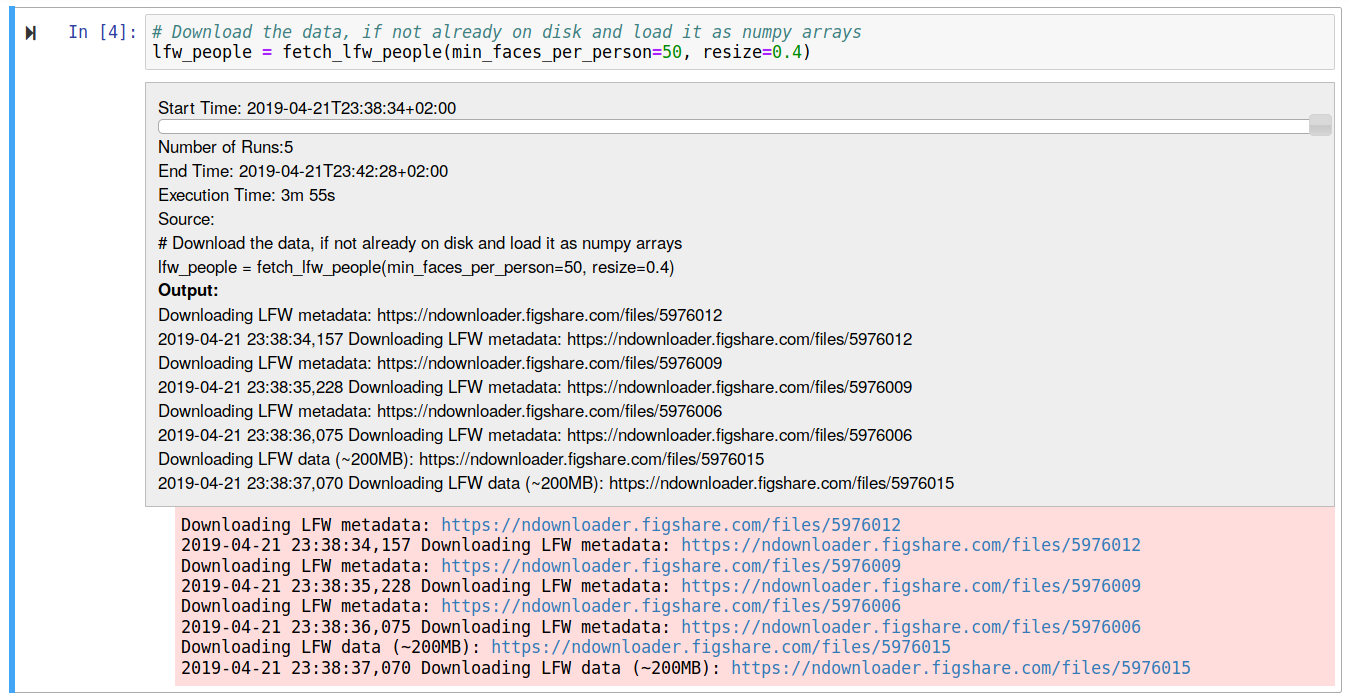

Supplement: Supplemental Information 2 — Each figure shows the ProvBook in different scenarios. It contains the provenance difference in different runs and execution environment. [file peerj-cs-08-921-s002.zip › Evaluation/SupplementaryFile/ExecutionEnvironment_Ubuntu1804.png]

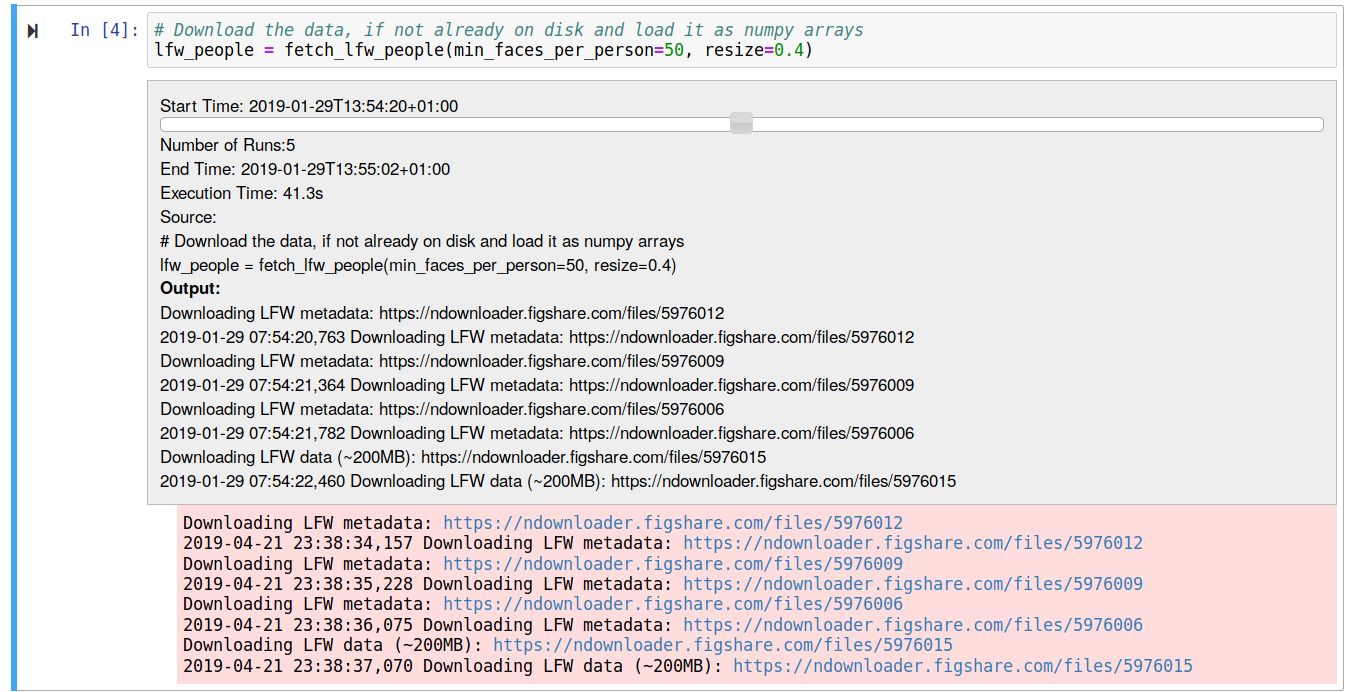

Supplement: Supplemental Information 2 — Each figure shows the ProvBook in different scenarios. It contains the provenance difference in different runs and execution environment. [file peerj-cs-08-921-s002.zip › Evaluation/SupplementaryFile/ExecutionEnvironment_Ubuntu1810.png]

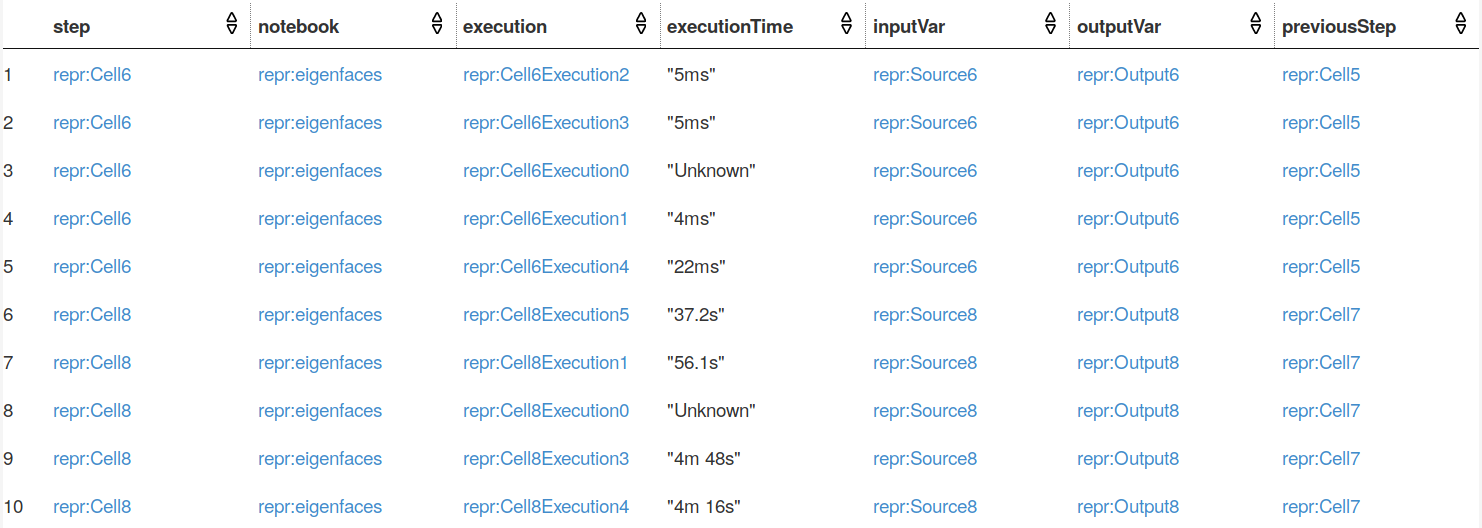

Supplement: Supplemental Information 2 — Each figure shows the ProvBook in different scenarios. It contains the provenance difference in different runs and execution environment. [file peerj-cs-08-921-s002.zip › Evaluation/SupplementaryFile/Listing2Result.png]
