# Supplementary material for: A collaborative semantic-based provenance management platform for reproducibility
Source: PeerJ Comput Sci. 2022 Mar 10;8:e921. doi: 10.7717/peerj-cs.921 (PMC9044346; doi:10.7717/peerj-cs.921)
Supplement: Supplemental Information 3 — The purpose of this questionnaire was to see how the users find CAESAR useful with respect to the features it provides. [file peerj-cs-08-921-s003.pdf]

CAESAR (CollAborative Environment for Scientific Analysis with Reproducibility) Evaluation

1. **Please rate the perceived usefulness of CAESAR.**

	**Strongly Agree**	**Agree**	**Neither agree nor disagree**	**Disagree**	**Strongly disagree**
It enables me to organize my experimental data more efficiently					
Preserving data in CAESAR helps the new comers in the project to understand the ongoing work in the team					
It helps me to search all the data related to my experiments including images, their metadata and device settings					
It enables a collaborative environment among my team members					
It enables me to visualize all the experimental data and results effectively					
It enables me to link the images to the experimental data and results					

2. **Please rate the following questions in regard to your experience with CAESAR.**

	**Strongly Agree**	**Agree**	**Neither agree nor disagree**	**Disagree**	**Strongly disagree**
CAESAR is useful for your scientific data management					
CAESAR is user-friendly					
CAESAR provides a collaborative environment among teams					
It is easy to learn to use it					

3. **What do you think about the following features in CAESAR?**

	**Strongly Like**	**Like**	**Neither like nor dislike**	**Dislike**	**Strongly dislike**
Project Dashboard (An one-place overview of all the experiments for a project)					
ProvTrack (A visualization module to track the experimental data including the link between images, experiments and metadata)					
ProvBook (A computational Reproducibility framework for data analysis scripts in Jupyter Notebook)					

4. **Please let us know the overall feedback of CAESAR along with its positive aspects and the things to improve.**
