# Supplementary material for: A collaborative semantic-based provenance management platform for reproducibility
Source: PeerJ Comput Sci. 2022 Mar 10;8:e921. doi: 10.7717/peerj-cs.921 (PMC9044346; doi:10.7717/peerj-cs.921)
Supplement: Supplemental Information 4 — The purpose of this study was to see how the users find CAESAR useful with respect to the features it provides. [file peerj-cs-08-921-s004.pdf]

CAESAR (CollAborative Environment for Scientific Analysis with Reproducibility) User Evaluation Responses

These are the responses of the participants of this study. The number in each row of the table denotes the number of participants who selected each category.

1. **Please rate the perceived usefulness of CAESAR.**

	Strongly Agree	Agree	Neither agree nor disagree	Disagree	Strongly disagree
It enables me to organize my experimental data more efficiently	2	4	0	0	0
Preserving data in CAESAR helps the new comers in the project to understand the ongoing work in the team	4	2	0	0	0
It helps me to search all the data related to my experiments including images, their metadata and device settings	4	2	0	0	0
It enables a collaborative environment among my team members	2	4	0	0	0
It enables me to visualize all the experimental data and results effectively	3	2	0	1	0
It enables me to link the images to the experimental data and results	2	4	0	0	0

2. **Please rate the following questions in regard to your experience with CAESAR.**

	Strongly Agree	Agree	Neither agree nor disagree	Disagree	Strongly disagree
CAESAR is useful for your scientific data management	2	3	0	0	0
CAESAR is user-friendly	0	3	2	0	0
CAESAR provides a collaborative environment among teams	0	5	0	0	0
It is easy to learn to use it	0	2	3	0	0

3. **What do you think about the following features in CAESAR?**

	**Strongly Like**	**Like**	**Neither like nor dislike**	**Dislike**	**Strongly dislike**
Project Dashboard (An one-place overview of all the experiments for a project)	1	3	1	0	0
ProvTrack (A visualization module to track the experimental data including the link between images, experiments and metadata)	3	2	0	0	0
ProvBook (A computational Reproducibility framework for data analysis scripts in Jupyter Notebook)	2	1	2	0	0

4. **Please let us know the overall feedback of CAESAR along with its positive aspects and the things to improve.**

   - CAESAR provides a lot of features. Therefore, it is difficult to follow them.
   - I find ProvTrack and ProvBook very useful among all the features in CAESAR. Sharing data among team members becomes easy with it.
   - CAESAR has the potential to be a valuable addition to the "Materials & Methods" section of a scientific publication. It makes it easy to find the resources used in an experiment by simple "clicking" via the many connections between the elements in the database, so that it is much clearer how a measurement was produced. This is also very useful for the internal organization of a research group as CAESAR enables e.g. new lab members to get a better overview over the experimental workflow. The main issue is the stability of the connection to the server. This is especially the case with bigger files.
