# Supplementary material for: A collaborative semantic-based provenance management platform for reproducibility
Source: PeerJ Comput Sci. 2022 Mar 10;8:e921. doi: 10.7717/peerj-cs.921 (PMC9044346; doi:10.7717/peerj-cs.921)
Supplement: Supplemental Information 5 — The document provides the supplementary information on the provenance visualization and representation modules. The panels of the Dashboard are explained in detail. The main classes of CAESAR database is provided. [file peerj-cs-08-921-s005.pdf]

CAESAR User Manual

Provenance Visualization - Dashboard Panels

The Plot: It displays the plot of an experiment by providing information on the Research Project, Research Group of the user the experiment belongs to, and the date of execution of the experiment.
The Characters: It provides information on all the agents that are involved in an experiment directly or indirectly.
It shows the name of the person, the experiment that the person is associated with, the step at which the agent was responsible, and the role of the person in the experiment.
Materials: It provides information on all the materials that are associated with an experiment.
It also shows the step at which the materials were referenced.
Additional properties of the materials are also provided in the table.
External Resources: It provides information on all the external resources that were referenced during the experiment lifecycle. These include the publications, files, or other external annotations used in the experiment.
Files: The panel provides information on all the files that were referenced during the experiment. It includes the detail about the step at which these files were used.
Jupyter Notebooks: It provides information on all the computational processes involving Jupyter Notebooks or scripts.
The name of the computational notebooks that are used in the experiment and the step at which they were used are displayed in the table.
Steps/Activities: It provides a list of all steps and activities that are associated with an experiment.
Devices: It shows all the devices used in the experiment along with their settings.
This information is extracted from the images and the experimental data.
Settings: It shows all the settings of the devices used in the experiment.
It includes the settings that were made during the experiment.
Results: It shows the results of a scientific experiment.
It includes the final and intermediate results.

Provenance Representation – Database Schema

A Project is a collection of Datasets.
A Dataset, which is a group of images, can belong to one or more Projects.
An Image represents the actual image with its metadata and can belong to one or more Datasets.
The device which is used to capture the Image is described by an Instrument.
The Instrument model consists of various components like Microscope, LightSource, Detector, Objective and Filters.
Each of these components provides description of its ManufacturerSpec and Settings.
The Experimenter class represents the person who is performing the imaging experiment.
The StructuredAnnotations composes of unordered collection of annotations that are attached to the objects like Project, Dataset or Image.
These StructuredAnnotations are of different types like XMLAnnotation, FileAnnotation, ListAnnotation, LongAnnotation, DoubleAnnotation, CommentAnnotation, BooleanAnnotation, TimestampAnnotation, TagAnnotation, TermAnnotation and MapAnnotation.
The Experiment class provided by OMERO will not suffice our requirements for capturing provenance information because this class only describes the type of experiment and the optional description field.
It consists of temporal and spatial information, the research group, the project and all the images captured during an experiment.
Each Dataset contains only one Experiment and has a one-to-one relationship.
The materials used in an experiment is added as classes like Plasmid, Protein, Vector, ChemicalSubstance, DNA, RNA, Amplifications, FluorescentProtein, Oligonucleotide and RestrictionEnzyme.
The model consists of StandardOperatingProcedure that describes the protocols used in an Experiment.
